# Transformative change to address biodiversity loss is urgent and possible

**DOI:** 10.1371/journal.pbio.3003387

**Published:** 2025-09-30

**Authors:** Anne Larigauderie, Karen O’Brien, Lucas A. Garibaldi, Arun Agrawal

**Affiliations:** 1 Intergovernmental Platform on Biodiversity and Ecosystem Services, Bonn, Germany; 2 Department of Sociology and Human Geography, University of Oslo, Oslo, Norway; 3 Universidad Nacional de Río Negro, Instituto de Investigaciones en Recursos Naturales, Agroecología y Desarrollo Rural, Río Negro, Argentina; 4 Consejo Nacional de Investigaciones Científicas y Técnicas, Instituto de Investigaciones en Recursos Naturales, Agroecología y Desarrollo Rural, Río Negro, Argentina; 5 Keough School of Global Affairs, University of Notre Dame, Notre Dame, Indiana, United States of America

## Abstract

Transformative change for a just and sustainable world often appears overwhelming. This Perspective highlights the key messages from the IPBES Transformative Change Assessment and how everyone can be involved in the strategies and actions needed to achieve transformative change.

## Teaser

Transformative change for a just and sustainable world often seems overwhelming, but it is possible. The IPBES Transformative Change Assessment shows how we can all be involved in the strategies and actions needed to achieve it.

Our planet is losing biodiversity at an unprecedented scale, with devastating consequences for people [1]. Yet, global efforts to stem biodiversity loss and the decline of nature have failed repeatedly. Instead, increasing levels of overconsumption and waste, coupled with the direct drivers of land-use and sea-use change, direct exploitation of organisms,  climate change, pollution, and invasive alien species have accelerated biodiversity loss during the past 50 years. The Intergovernmental Platform on Biodiversity and Ecosystem Services (IPBES) Global Assessment of Biodiversity and Ecosystem Services [1] concluded that transformative change is needed to achieve the Sustainable Development Goals and the 2050 Vision for Biodiversity.

Transformative change for a just and sustainable world encompasses fundamental, system-wide shifts in views (ways of thinking, knowing, and seeing), structures (ways of organizing, regulating, and governing), and practices (ways of doing, behaving, and relating) to address the underlying causes of biodiversity loss and nature’s decline. The recently approved Kunming-Montreal Global Biodiversity Framework recognizes the importance of transformative change to achieve the 2050 Vision for Biodiversity in aiming “to catalyze, enable and galvanize urgent and transformative action” by all [2]. The recently completed IPBES Transformative Change Assessment aims to inform the implementation of the Global Biodiversity Framework [3]. It was prepared by an interdisciplinary group of ~100 scientists and holders of Indigenous and local knowledge. They worked for over 3 years, critically assessing ~7,000 publications and drawing on the expertise of an additional 200 contributing authors. Analysis of 881 shared positive visions of desirable futures and ~400 real-world case studies provided both aspirational and empirical insights into transformative change. The Report was approved by the 11th session of the Plenary of IPBES in December 2024.

Drawing on the IPBES Transformative Change Assessment Report [3], we explain why past efforts to address biodiversity loss have been ineffective, outline five key strategies to achieve the 2050 Vision for Biodiversity, and highlight the roles we can each play in these strategies.

**Fig 1:**
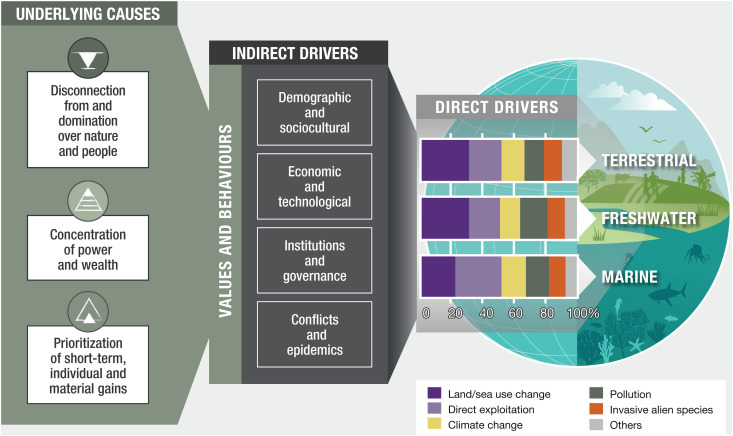
Underlying causes, indirect drivers and direct drivers of biodiversity loss and nature’s decline. This figure shows how the IPBES Transformative Change Assessment specifies and synthesizes the key underlying causes that underpin, cut across, shape, and reinforce the indirect and direct drivers of biodiversity loss and nature’s decline. The figure builds on figure SPM.2 of the Summary for Policymakers of the Global Assessment Report on Biodiversity and Ecosystem Services, including its identification of indirect and direct drivers, with the latter represented in the bar chart showing the proportional contributions of each direct driver to biodiversity loss in terrestrial, freshwater, and marine ecosystems. Further details on the analysis leading to the identification of these indirect and direct drivers and on the calculation of contributions to biodiversity loss across different ecosystems can be found in the IPBES Global Assessment Report on Biodiversity and Ecosystem Services [1]. More information on the underlying causes and how they manifest across views, structures, and practices (including values and behaviors) is provided in the Transformative Change Assessment [3]. This figure corresponds to figure SPM.1 of the Summary for Policymakers of the IPBES Transformative Change Assessment [3], and is reproduced with permission.

Past efforts to stem biodiversity loss and nature’s decline have not met their goals because they have failed to address the underlying causes: deeply rooted, interconnected social and cultural patterns that shape, influence, and reinforce direct and indirect drivers of biodiversity loss. Three underlying causes present persistent challenges and barriers to transformative change (Fig 1). First, are the disconnection from and domination over nature and people. These lead to views, structures, and practices that drive unsustainable use and exploitation of nature, as well as the appropriation of land and labor of marginalized peoples and communities for economic gain. Second, is the increasing concentration of power and wealth, which supports the interests of a small number of people whose actions disproportionately drive biodiversity loss and nature’s decline. In 2021, the share of global wealth for the top 1% of the global population was 39.2%, while the bottom 50% owned 1.85% [3,4]. Third, is the prioritization of short-term, individual, and material gains, which places the immediate interests and desires of individuals before those of the community and against long-term social and ecological sustainability. These patterns are perpetuated through economic and social systems that equate human well-being, satisfaction, and happiness with the accumulation of material possessions.

Together, these three underlying causes contribute to persistent, systemic challenges and barriers to transformative change. Such challenges and barriers include economic and political inequalities, and unsustainable consumption and production patterns. Principles of transformative change, such as equity and justice, pluralism and inclusion, respectful and reciprocal human–nature relationships, and adaptive learning and action are important for addressing these challenges. Such principles can be applied to five key strategies and actions to advance transformative change towards a just and sustainable world.

The first strategy for transformative change emphasizes the conservation, restoration, and regeneration of places with both cultural and ecological significance. Biocultural approaches, such as community-led conservation, can simultaneously sustain ecosystems and strengthen cultural identity, particularly when Indigenous and local governance systems are respected and empowered.

The second strategy focuses on the integration of biodiversity concerns into the sectors most responsible for environmental degradation. These sectors include agriculture, fisheries, forestry, infrastructure, and energy. Agroecological transitions are an important example to enhance food security, restore biodiversity, and empower small-scale farmers (and thereby to increase equity). Similarly, technological innovations, such as those in renewable energy or financial tools, can support nature if guided by principles of equity and sustainability. Reducing or redirecting harmful subsidies, which in 2023 amounted to up to $3.3 trillion per year [5], will close the global biodiversity finance gap while lowering pressures on ecosystems.

The third strategy calls for a transformation of economic and financial systems for nature and equity. This includes internalizing environmental costs, reforming fiscal tools, and shifting the metrics of success to account for ecological integrity and social equity. Changing the ways economies measure progress, from gross domestic product to inclusive indicators of well-being, can realign development with sustainability.

The fourth strategy emphasizes governance reforms. Inclusive, accountable, and adaptive governance is essential to build legitimacy and coordination across scales and actors. Institutional arrangements that foster shared planning, mutual accountability, and respect for diverse knowledge systems support transformative outcomes and better navigate trade-offs.

Finally, shifting societal views and values is key. Recognizing the interconnectedness of people and nature through education, communication, and social movements can reshape norms and behaviors. Curricula that integrate systems thinking, nature appreciation, and empathy help to educate new generations that are prepared to create and thrive in a just and sustainable world. Embracing Indigenous knowledge and co-creating new social imaginaries further strengthens this transition.

By influencing ways of thinking, governing, and acting to address underlying causes of biodiversity loss, these five strategies and associated actions can change entrenched patterns and unsustainable pathways. Collectively, they constitute viable mechanisms for transformative change to achieve the Sustainable Development Goals and the 2050 Vision for Biodiversity.

Based on the IPBES Transformative Change Assessment [3], we emphasize that every person, group, and organization has a role in pursuing the actions needed to create transformative change. Even small, incremental changes can contribute to transformative change when they enable shifts in views, structures, and practices that address the underlying causes of biodiversity loss and nature’s decline. The quality and direction of incremental changes is of critical importance to amplify and scale transformative change across levels, sectors, and systems. Governments are powerful enablers of transformative change when they foster policy coherence, enact and enforce stronger regulations to benefit nature and nature’s contributions to people in policies and plans across different sectors, deploy innovative economic and fiscal tools, phase out harmful subsidies, and promote international cooperation. Actions by businesses, including sustainable supply chains, voluntary disclosures, and commitments to engage with Indigenous Peoples and local communities and small producers, are ways to promote sustainability solutions. Civil society organizations have mobilized citizens, held governments and the private sector accountable, fueled public debates, and innovated new scalable models for sustainability. An analysis of 2,802 mobilizations between 1992 and 2023, for example, provides evidence that they contested 46,955 documented environmental threats [5]. Case studies reveal the power and potential of actor coalitions, including scientists, in realizing transformative potential.

Transformative change is urgently needed. Without it, repeated failures to reach global sustainability and biodiversity goals will continue to occur because they do not attend to the underlying causes of biodiversity loss and trajectories of unsustainable change. The Transformative Change Assessment from IPBES emphasizes that everyone has a role to play in the strategies and actions needed to achieve the 2050 Vision for Biodiversity. Indeed, the findings of the Assessment are an urgent call to action and a source of knowledge for concerted efforts across scales to pursue transformative change towards a just and sustainable world.

## Abbreviations

IPBES: Intergovernmental Platform on Biodiversity and Ecosystem Services
